# Bleomycin reduces *Vairimorpha (Nosema) ceranae* infection in honey bees with some evident host toxicity

**DOI:** 10.1128/spectrum.03349-23

**Published:** 2024-01-05

**Authors:** Parker Parrella, Annabelle B. Elikan, Helen V. Kogan, Fatoumata Wague, Corey A. Marshalleck, Jonathan W. Snow

**Affiliations:** 1 Department of Biology, Barnard College, New York, New York, USA; Agroscope, Nyon, Switzerland

**Keywords:** *Vairimorpha*, *Nosema*, microsporidia, experimental therapeutics, honey bee, infection, bleomycin

## Abstract

Microsporidia cause disease in many beneficial insects, including honey bees, yet few pathogen control tools are available for protecting these important organisms against infection. Some evidence suggests that microsporidia possess a reduced number of genes encoding DNA repair proteins. We hypothesized that microsporidia would thus be susceptible to treatment with DNA-damaging agents and tested this hypothesis using a novel, rapid method for achieving robust and homogenous experimental infection of large numbers of newly emerged honey bees with one of its microsporidia pathogens, *Vairimorpha* (*Nosema*) *ceranae*. In carrying out these experiments, we found this novel *V. ceranae* inoculation method to have similar efficacy as other traditional methods. We show that the DNA-damaging agent bleomycin reduces *V. ceranae* levels, with minimal but measurable effects on honey bee survival and increased expression of midgut cellular stress genes, including those encoding SHSP. Increased expression of *UpdlC* suggests the occurrence of epithelial regeneration, which may contribute to host resistance to bleomycin treatment. While bleomycin does reduce infection levels, host toxicity issues may preclude its use in the field. However, with further work, bleomycin may provide a useful tool in the research setting as a potential selection agent for genetic modification of microsporidia.

**IMPORTANCE:**

Microsporidia cause disease in many beneficial insects, yet there are few tools available for control in the field or laboratory. Based on the reported paucity of DNA repair enzymes found in microsporidia genomes, we hypothesized that these obligate intracellular parasites would be sensitive to DNA damage. In support of this, we observed that the well-characterized DNA damage agent bleomycin can reduce levels of the microsporidia *Vairimorpha* (*Nosema*) *ceranae* in experimental infections in honey bees. Observation of slightly reduced honey bee survival and evidence of sublethal toxicity likely preclude the use of bleomycin in the field. However, this work identifies bleomycin as a compound that merits further exploration for use in research laboratories as a potential selection agent for generating genetically modified microsporidia.

## INTRODUCTION

Microsporidia are obligate intracellular parasites that cause widespread infections in nature but are relatively understudied compared to microbial pathogens representing other taxonomic groups such as bacteria and non-microsporidia fungi (1). *Vairimorpha* (*Nosema*) *ceranae* is a microsporidian parasite that is pathogenic to honey bees, and infection by these species has been implicated as a key factor in honey bee losses (2, 3). In the United States, *V. ceranae* infection has traditionally been treated with the MetAP2 inhibitor fumagillin, but the use of this drug is prohibited in Europe [reviewed in reference (4)] and its efficacy in controlling *V. ceranae* in the field is uncertain (5). Additionally, fumagillin may be detrimental to host cell function at high doses, *V. ceranae* may be able to evade suppression in some circumstances (6), and recent availability of fumagillin has been inconsistent. Many promising alternative strategies for the mitigation of *V. ceranae* infection are now being pursued [see reference (7) and references therein]. Reduced genome sizes that represent a defining feature of microsporidia as a phylogenetic group. As such, one therapeutic approach seeks to take advantage of reduced pathogen resilience at the cellular level conferred by this genome compaction.

The microsporidia *Encephalitozoon cuniculi* were found to possess a dramatic reduction in the number of identifiable genes from the double-strand break repair pathways (8). This paucity in double-strand break repair pathways has been shown to be generalizable to other microsporidia genomes (9), including that of *V. ceranae* [although see reference (10)]. We therefore hypothesized that *V. ceranae* would be susceptible to treatment with DNA-damaging agents. To test this hypothesis, we first characterized a novel method of achieving rapid, robust, and homogenous experimental infection of large numbers of newly emerged bees with *V. ceranae*. We then selected bleomycin as a DNA-damaging agent due to the wealth of knowledge about its mechanism of action, its well-characterized effects in prokaryotic and eukaryotic cells (11), its widespread use as a selection agent in diverse cell types, and a comprehensive understanding of its impact on insects (12).

## MATERIALS AND METHODS

### Honey bee colonies and caging experiments

Honey bees were collected from outbred colonies in New York, NY, USA consisting of a typical mix of *Apis mellifera* subspecies found in North America, at different times during the months of April–October. Source colonies were visually inspected for symptoms of common bacterial, fungal, and viral diseases of honey bees. For caging experiments, bees were collected from the landing board, or newly emerged bees were collected after hatching from a capped brood frame overnight. Unless otherwise stated, approximately 25 landing board bees or ~65 newly eclosed bees were placed into each 12.2 × 8.6 × 21.3 cm acrylic cage with sliding door machined at Carleton Labs, Columbia University. For cages containing newly eclosed bees, approximately four foragers from the same source colony [marked with a spot of paint (Testors, Vernon Hills, IL, USA)] were added to each cage. Caged bees were maintained in incubators at 35°C (unless otherwise stated) in the presence of PseudoQueen (Contech, Victoria, BC, Canada) added to each incubator as a source of queen mandibular pheromone.

### *Vairimorpha ceranae* and spore isolation and quantification

*V. ceranae* spores were obtained from infected individuals and serially passaged through bees as performed previously [see references (7, 13)] to provide spores for infection experiments. To isolate spores, midguts from infected or uninfected bees were individually crushed in 0.5 mL of H_2_O with a pestle, and the spore number was assessed by light microscopy. Homogenized midguts were washed with water and resuspended in 33% sucrose solution at a concentration of 2 × 10^6^ spores/mL for infection experiments.

### *Vairimorpha ceranae* infections and chemical treatments

For *V. ceranae* infections by group feeding method, newly eclosed bees caged as above were allowed to consume sucrose solution containing spores (2 × 10^6^/mL as indicated) *ad libitum* for 48 hours before food was replaced with sucrose solution alone. For *V. ceranae* infections by the individual feeding method, newly eclosed bees were immobilized and individually fed a single droplet (2 µL) of sucrose solution containing 2 × 10^6^ spores/mL (each bee received ~4,000 spores). Bees were then transferred to cages as above and fed sucrose solution alone for the remainder of the experiment. The novel soak method is based on a method described by Leonard et al. using a “soaking” inoculation to introduce genetically engineered, *Snodgrassella alvi* into newly eclosed bees (14). For *V. ceranae* infections by the soak method, a 50 mL conical tube was filled to the 25 mL mark with newly eclosed bees (~65 bees). Sucrose solution (1 mL) containing *V. ceranae* spores (2 × 10^6^/mL as indicated) was added to the conical tube, which was mixed by gentle inversion at 5-minute intervals for a total of 20 minutes of exposure. Bees were then transferred to cages as above and fed sucrose solution alone for the remainder of the experiment. When the soak method was used for larger groups of bees for bleomycin treatments, up to 100–150 mL of newly eclosed bees were infected in a 400 mL glass beaker using 3.5 mL of sucrose solution containing *V. ceranae* spores (2 × 10^6^/mL) with a similar time as above before being split into cages with ~65 bees per cage.

For chemical treatments on infected bees, honey bees infected by the soak method (see above) were fed sucrose solution alone or sucrose solution containing bleomycin at the indicated dose (in a range from 0.153 to 1.25 µg/mL) beginning at 6 days post-infection. After 4 days of drug feeding, honey bee midguts were dissected, crushed in 0.5 mL of water, and the number of mature spores was counted by light microscopy as previously described (7) unless otherwise stated. In parallel, quantitative PCR (qPCR) was used to determine the relative amount of *V. ceranae* genome equivalents versus host genome equivalents.

For survival experiments and gene expression analysis on uninfected bees, newly emerged bees were caged and fed as above. For survival, bees were switched to sucrose solution alone or sucrose solution containing bleomycin at the indicated dose (0.153, 0.306, 0.613, 1.25, 2.5, 6.25, 25, or 100 µg/mL) for 10 days starting on 4 days post-eclosion while survival was assessed. For biomarker gene expression, bees were switched to sucrose solution alone or sucrose solution containing bleomycin at 0.613 µg/mL for 4 days starting on 6 days post-eclosion prior to dissection and gene expression analysis.

### DNA extraction and qPCR

DNA extraction was performed using a modified Smash and Grab DNA Miniprep protocol as described previously (13). The resulting DNA was used as a template for qPCR to determine the level of infection for *Vairimorpha* sp. using primers for the *Vairimorpha apis 16S* gene and the *V. ceranae β-actin* relative to the honey bee *ATP5a* gene (15, 16). For qPCR reactions, PowerUp SYBR Green Master Mix (Applied Biosystems, Foster City, CA, USA) was used in accordance with the manufacturer’s instructions in a LightCycler 480 thermal-cycler (Roche, Branchburg, NJ, USA) or Bio-Rad CFX Opus (Bio-Rad, Hercules, CA, USA). The difference between the threshold cycle (Ct) number for honey bee *ATP5a* and that of the *Vairimorpha* sp. of interest was used to calculate the relative level of infection using the 2^(-ΔCT)^ method (17). A sample was considered negative for a specific *Vairimorpha* species if it did not amplify any product by 35 cycles and zero was entered as the value in these cases. For examining levels of total bacteria and one specific bacterial species of the digestive tract microbiome (*Gilliamella apicola*), a similar assay was performed using universal 16S rRNA primers and species-specific 16S rRNA primers from reference (18) in conjunction with the honey bee *ATP5a* gene. *G. apicola* was chosen as the single bacterial species to investigate in more detail because previous studies have found that this is the predominant species in uninoculated cage-reared bees (19, 20), while other bacterial subsets are typically highly variable and at very low levels.

### RNA isolation, reverse transcription, and quantitative PCR for gene expression analysis

RNA was prepared from bees’ midgut tissue as previously described (21). Midgut tissue was manually crushed with a disposable pestle in Trizol Reagent (Invitrogen, San Diego, CA, USA), and RNA was then extracted as per the manufacturer’s instructions. RNA was then DNase treated using RQ1 RNase-Free DNase I (Promega, Madison, WI, USA) and cDNA was synthesized using approximately 1 µg of RNA with the High-Capacity cDNA Reverse Transcription Kit with RNase Inhibitor (Applied Biosystems, Foster City, CA, USA). For qPCR reactions to determine the expression levels of genes of interest, 1 µL of cDNA was used as a template in conjunction with PowerUP SYBR Green Master Mix (Applied Biosystems, Foster City, CA, USA) and appropriate primers in a 20 µL reaction. Reactions were run in a LightCycler 480 thermal cycler (Basel, Switzerland) or Bio-Rad CFX Opus (Bio-Rad, Hercules, CA, USA). Primer sequences targeting transcripts of genes of interest are from references (21, 22). The difference between the threshold cycle number for *β-actin* and that of the gene of interest was used to calculate the level of that gene relative to *β-actin* using the typical 2^(-ΔCT)^ method (17). All qPCR data represent expression values from individual bees (sample sizes found in figure legends) and is displayed as mean ± SEM.

### Total lysate and Western blot analyses

For the phosphorylated histone Western blot, landing board bees were fed sucrose solution containing 2.5 or 25 µg/mL bleomycin for 48 hours or sucrose solution alone. Total lysates from five pooled midguts were generated by pestle-mediated homogenization of remaining midgut tissues in RIPA buffer containing protease inhibitors (Sigma, St. Louis, MO, USA) and phosphatase inhibitors (Halt Phosphatase Inhibitor, Thermo Scientific, Waltham, MA, USA). Following incubation on ice to allow for cellular lysis, lysates were centrifugated to remove cellular debris. For Western blot analysis, aliquots of total lysates containing 50 µg of total protein were fractionated on an SDS-polyacrylamide gel and electroblotted onto polyvinylidene difluoride membrane. Antibody incubation and chemiluminescence detection (using the Chemiluminescent Peroxidase Substrate-3 kit) were performed according to the manufacturer’s instructions (Sigma, St. Louis, MO, USA). The antibodies used include those directed to GAPDH (HRP-60004, Proteintech, Rosemont, IL, USA) directly conjugated to HRP and pHistone H2AvD (Ser137) (600-401-914, Rockland Immunochemicals Pottstown, PA, USA) followed by use of Goat anti-rabbit secondary conjugated to HRP. Western blot visualization was performed using a Bio-Rad ChemiDoc MP Imaging System (Bio-Rad, Hercules, CA, USA).

### Statistical analysis

Data are presented as means ± SEM. For two groups, data were compared using unpaired *t*-tests with Welch’s correction when values fit normal distributions or Mann-Whitney *U* nonparametric tests when they did not fit normal distributions. Normality was assessed using Shapiro–Wilk tests. When more than two groups were being compared, data were compared using one-way ANOVA with Tukey’s multiple comparison test when values fit normal distributions or a Kruskall-Wallis test when they did not. For survival analysis, treated versus untreated groups were compared using the Mantel-Cox test. Unless otherwise stated, all experiments were performed a minimum of three independent times.

## RESULTS

### Novel soak inoculation method produces rapid, robust, and homogenous *V. ceranae* infection in newly emerged honey bees

**Fig 1:**
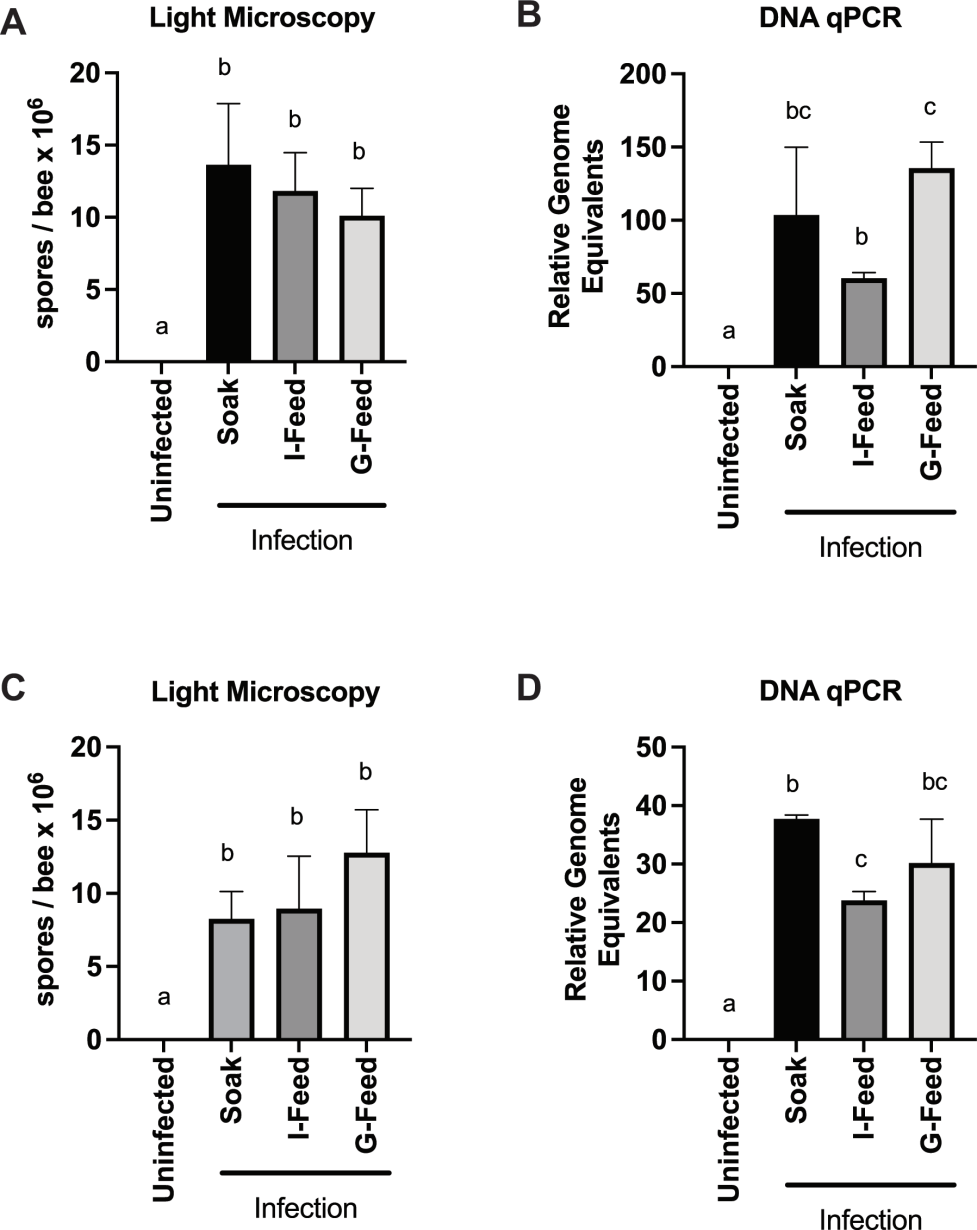
Soak method produces *V. ceranae* infection levels comparable to traditional methods of inoculation in newly eclosed bees. *V. ceranae* levels in midguts of newly eclosed bees left uninfected or inoculated via the group feeding method, the individual feeding method, or the soak method on day 8 post-infection as determined by spore count using light microscopy (**A**) or by qPCR (**B**) and on day 14 post-infection as determined by spore count using light microscopy (**C**) or by qPCR (**D**) (Uninfected: *n* = 10, Soak: *n* = 10, I-Feed: *n* = 10, and G-Feed: *n* = 10) *a* ≠ *b*, *P* < 0.05.

We first compared the novel soak method with traditional group feeding and individual feeding methods in newly eclosed bees (16). Honey bee midguts were dissected on day 8 and day 14 post-inoculation and infection levels were assessed by spore counting and qPCR. We observed high levels of infection comparable to the traditional group feeding and traditional individual feeding methods on both day 8 (Fig. 1A and B) and day 14 (Fig. 1C and D) post-infection. We examined a time course of infection using the soak method and found minimal spores on day 6 post-infection, with spore levels above the limits of detection in only 58% of bees. By contrast, on days 10, 14, and 18 post-infection, we observed 100% prevalence and high spore levels (approximately 100-fold higher average compared to day 6) in soak-infected bees (Fig. S1A), similar to infection time courses reported for other methods. To determine whether there was experiment-to-experiment variation in spore levels, we performed 30 soak experiments over the course of a season and examined spore levels 10 days post-infection. We found that there was considerable variability between experiments (Fig. S1B). However, when we looked at cage-to-cage variability in an experiment by moving soak-infected bees into separate cages immediately following infection, we observed no difference in spore levels between cages 10 days post-infection (Fig. S1C).

### Bleomycin reduces *V. ceranae* infection intensity in honey bees

**Fig 2:**
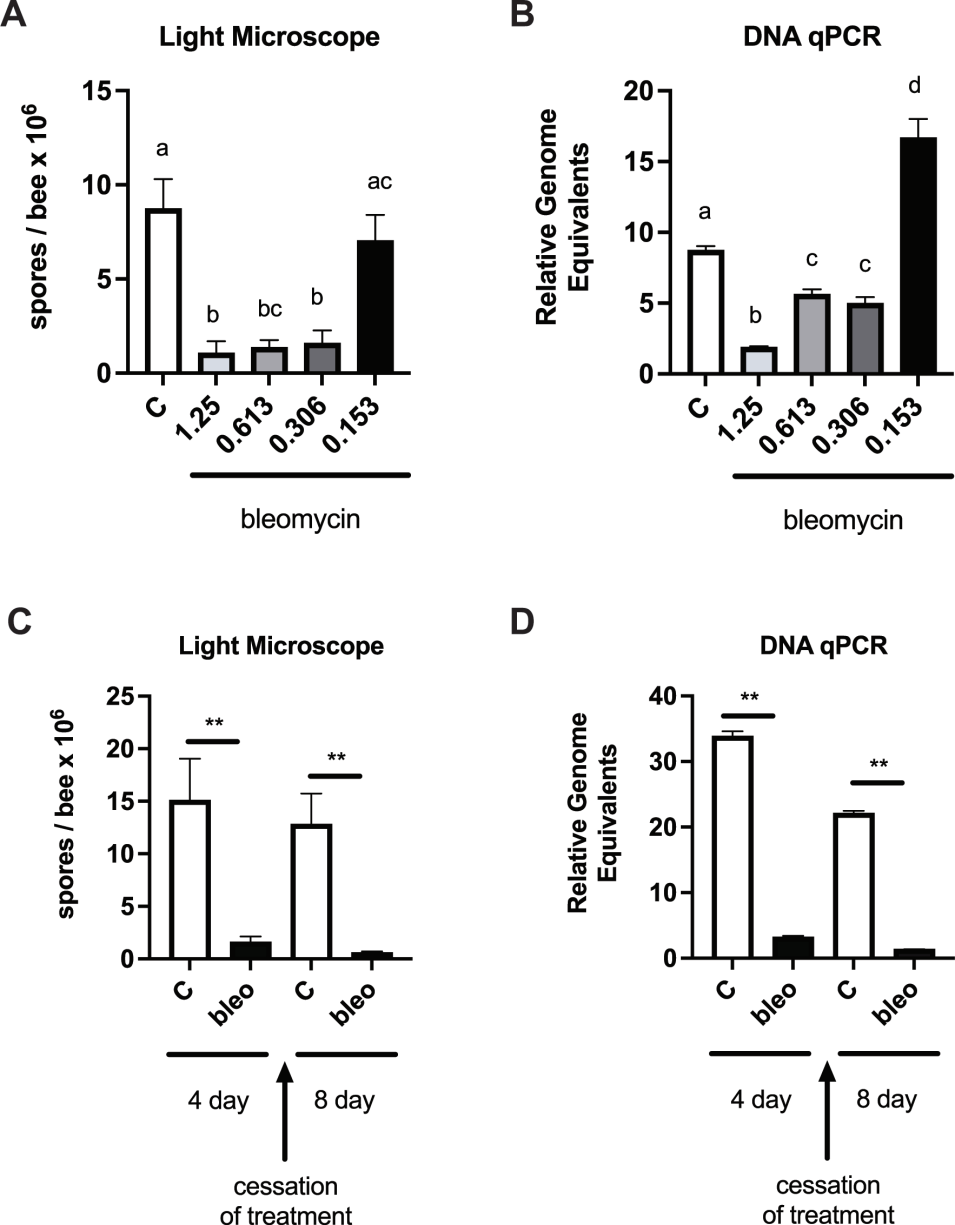
Bleomycin treatment reduces *V. ceranae* infection level in newly eclosed bees. *V. ceranae* levels in midguts of infected newly eclosed bees fed sucrose syrup with or without bleomycin at the indicated doses for 8 days as determined by spore count using light microscopy (**A**) or by qPCR (**B**) (C: *n* = 9, 1.25: *n* = 8, 0.613: *n* = 8, 0.306: *n* = 8, and 0.153 *n* = 8). *V. ceranae* levels in midguts of infected newly eclosed bees fed sucrose syrup or sucrose syrup containing 0.613 µg/mL bleomycin for 4 days (C: *n* = 11 and 0.613: *n* = 10) before switching to sucrose solution alone for 4 additional days (C: *n* = 10 and 0.613: *n* = 10) as determined by spore count using light microscopy (**C**) or by qPCR (**D**) *a* ≠ *b*, *P* < 0.05.

Bleomycins are a family of naturally occurring non-ribosomal glycopeptides, produced by *Streptomyces verticillus*, which has activity against both prokaryotic and eukaryotic cells by induction of DNA strand breaks (11). To test the effects of bleomycin (structure shown in Fig. S2A) on *V. ceranae* infection, newly eclosed bees were infected via the novel soak method described above. On day 6 post-infection, bees were treated with bleomycin at the indicated doses. Honey bee midguts were dissected at 4 days of drug feeding, and infection levels were assessed by spore counting and qPCR (13). We observed reductions in infection levels as assessed by spore counting and relative genome equivalents after bleomycin treatment at doses as low as 0.306 µg/mL (Fig. 2A and B). No decrease in infection intensity was observed at 0.153 µg/mL.

To determine the durability of *V. ceranae* inhibition (6), we treated infected newly eclosed bees with either sucrose solution alone or sucrose solution containing bleomycin for 4 days. We then switched all cages to sucrose solution alone for 4 days. Infection intensity was measured by spore counting, and DNA showed that while infection level remained high for bees receiving sucrose solution for the whole experiment, it decreased for those bees fed bleomycin for 4 days and remained low even after switching bees to sucrose solution without drug (Fig. 2C and D). This suggests that even with a short treatment course, bleomycin can eliminate infection with no evidence of subsequent reemergence.

### Bleomycin has minimal but measurable effects on host survival at doses effective in reducing *V. ceranae*

**Fig 3:**
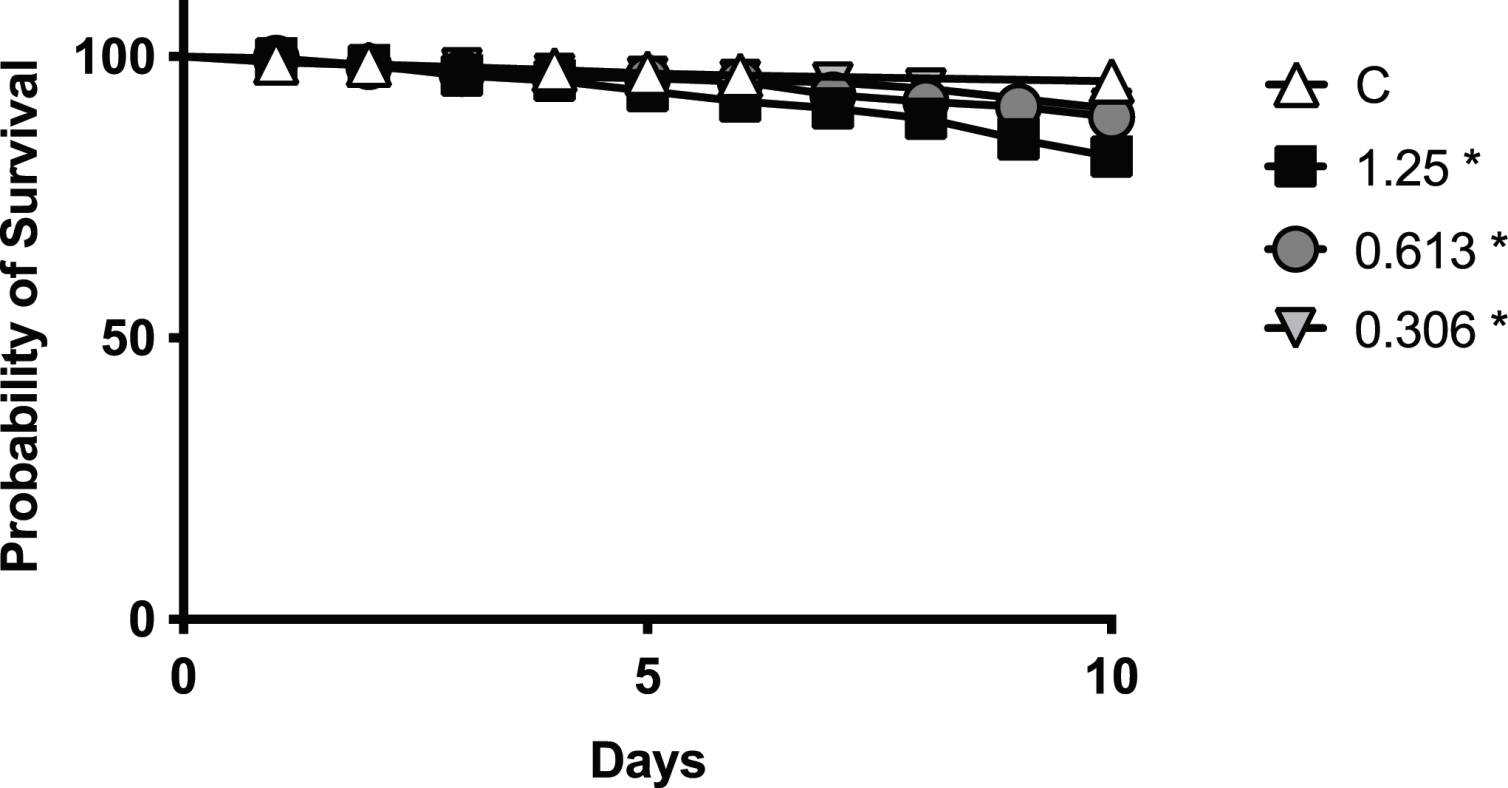
Bleomycin has minimal impacts on honey bee host survival. Survival of individual uninfected newly eclosed bees fed sucrose solution (*n* = 286) or bleomycin at 1.25 (*n* = 269), 0.613 (*n* = 301), or 0.306 (*n* = 177) µg/mL bleomycin starting on day 4 post-eclosion. The combined results from four independent trials are shown (*P < 0.05 relative to control).

Because bleomycin is lethal at high doses in the fruit fly *Drosophila melanogaster* [e.g., 25 µg/mL (12)], we explored the effects of bleomycin on honey bee survival. Newly emerged bees were fed sucrose solution alone or containing bleomycin at 100, 25, and 6.25 µg/mL for 10 days starting on 4 days post-eclosion. We found 100% mortality of bees in all bleomycin-fed groups at these high concentrations by 9 days post-treatment, while untreated bees had over 90% survival over 10 days (Fig. S2B, survival statistics can be found in Table S1A). In a second, high-dose, survival experiment, newly emerged bees fed sucrose containing bleomycin at 2.5 µg/mL for 10 days starting on 4 days post-eclosion had greater than 85% survival over this time period (Fig. S2C, survival statistics can be found in Table S1B). We then administered bleomycin at doses that were effective at reducing *V. ceranae* levels and examined the impact on honey bee survival (Fig. 3). Combining the data from four independent trials, we observed that bleomycin had moderate impacts on the survival when administered at 1.25 µg/mL (78.4% survival, Chi square: 27.21, df = 1, *P* ≤ 0.01), but only minimal (although statistically significant) impacts on the survival when administered at 0.613 µg/mL (88.0% survival, Chi square: 8.744, df = 1, *P* < 0.01) and 0.306 µg/mL (89.8% survival, Chi square: 4.671, df = 1, *P* = 0.0307) as compared to untreated bees (95% survival). There was some variability between the four trials as we observed no effects (three of four trials) or only small effects (one of four trials) on the survival of bees fed sucrose solution containing bleomycin at 0.613 µg/mL for 10 days starting on 4 days post-eclosion. Importantly, we observed no effects in any individual trial on the survival of bees fed sucrose solution containing bleomycin at 0.306 µg/mL for 10 days starting on 4 days post-eclosion (Fig. 3; Fig. S5; survival statistics can be found in Tables S1C through G).

### Bleomycin treatment increases the expression of stress biomarker genes and the regeneration-promoting *UpdlC* gene at doses effective in reducing *V. ceranae*

Although we did not observe a substantial and reproducible decrease in the survival of honey bees fed low doses (<1.25 µg/mL), understanding the extent of potential sublethal effects of this treatment is also important. We first examined the impact of bleomycin at high doses on a number of putative stress biomarkers to determine suitable markers to measure at lower, therapeutic doses of bleomycin. We first used Western blots to look for evidence of a histone marker (phosphorylated histone H2AvD at Ser137) characteristic of DNA damage response in other organisms, such as *Drosophila melanogaster* (12, 23). Pooled midgut samples from bees fed sucrose solution containing 2.5 or 25 µg/mL bleomycin showed a signal for phosphorylated histone, while pooled samples from bees fed sucrose solution alone did not (Fig. S3). All pooled samples showed a signal for the loading control GAPDH. However, the low sensitivity of this assay suggests that other metrics for measuring the sublethal effects of bleomycin on host cells should be explored.

Thus, we looked at a number of transcriptional targets known to be upregulated by tissue damage in bees. First, we examined the expression of select *shsp* genes of the *l(2)efl* family, which have been identified as useful stress biomarker genes in honey bees (13, 21, 24–26). Using qPCR, we found that expression of *724367* and *410087*a was increased in the midguts of uninfected bees treated with bleomycin (at 25 µg/mL) relative to control bees after 2 days of feeding (Fig. S4A and B). Bleomycin-induced damage to the insect digestive tract has been observed before and epithelial regeneration is a typical response (12), driven in part by increased production and release of cytokines of the UPD family (27) [and reviewed in reference (28)]. We find that at least one *Upd*-like gene, *UpdlC* [which we have recently characterized in bees (22)], is upregulated after 2 days of feeding with high dose bleomycin (Fig. S4C).

**Fig 4:**
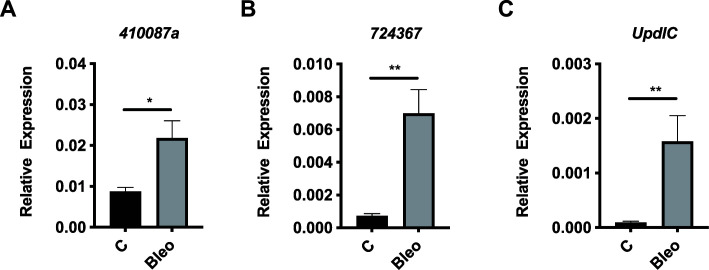
Bleomycin induces stress biomarker gene expression at doses that are effective at reducing *V. ceranae* infection intensity. Transcript levels of the *l(2)efl* genes *410087a* (**A**) and *724367* (**B**) and cytokine gene *UpdlC* (**C**) relative to the β-actin in midgut tissue from newly eclosed bees fed sucrose solution alone (*n* = 42) or sucrose solution containing 0.613 µg/mL (*n* = 42) for 4 days starting on day 6 post-eclosion (combined from five independent trials). Means ± SEM are shown and represent the expression values of the genes of interest, calculated using the 2^(-ΔCT)^ method for individual bees. Statistical significance is noted as * for *P* < 0.05 and ** for *P* < 0.01.

We then examined the sublethal effects of bleomycin on honey bee cells when administered at lower, therapeutic doses found to reduce *V. ceranae* infection. Combining the data from five independent trials, we observed induction of all three stress genes in bees treated with bleomycin at 0.613 µg/mL, relative to control bees (Fig. 4). However, there was again some variability between the five trials as we observed statistically significant expression changes for *410087*a in one of five trials, for *724367* in three of five trials, and *UpdlC* in two of five trials (Fig. S6).

### Bleomycin has no consistent impact on the host microbiome at doses effective in reducing *V. ceranae*

**Fig 5:**
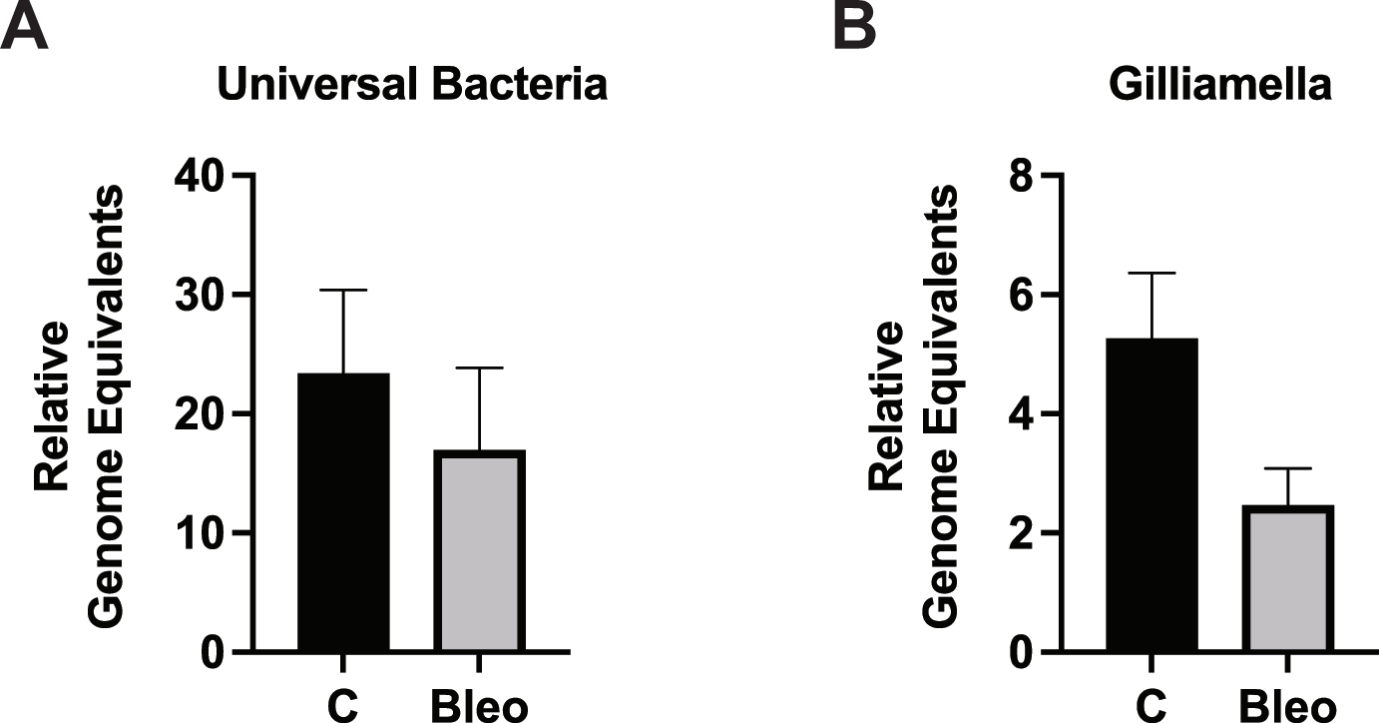
Bleomycin has no consistent impact on the host microbiome at doses effective in reducing *V. ceranae*. Levels of all bacteria (**A**) and the key microbiome community member *Gilliamella apicola* (**B**) as determined by qPCR in midguts of infected newly eclosed bees fed sucrose syrup alone (n=24) or containing 0.613 µg/mL (n=24) bleomycin for 4 days. The combined results from three independent trials are shown. Compared groups were not statistically significant.

To determine whether bleomycin treatment impacts the microbiome, we used primer sets amplifying the 16S rRNA region of all bacteria as well as sets that amplify the species-specific 16S rRNA region of the most predominant species in uninoculated cage-reared bees, *Gilliamella apicola* (19). Combining the data from three independent trials, we found that bleomycin (0.613 µg/mL) had no statistically significant effect on total bacteria levels or on the levels of *G. apicola* in the midgut (Fig. 5). Individual trials showed minor and variable effects on total bacteria levels and the levels of *G. apicola* in the midgut at the 0.613 µg/mL dose (Fig. S7).

## DISCUSSION

Experimental infection of honey bees by *V. ceranae* is critical for elucidating the important biology of this pathogen and its interaction with host cells and tissues. Traditional methods of inoculation involve either individual or group feeding approach [reviewed in reference (29)]. Individual inoculation uses hand delivery of a small defined volume of sucrose solution containing a known quantity of *V. ceranae* spores to immobilized bees often after a period of starvation [e.g., references (30–33)]. Group feeding uses *ad libitum* feeding of a group of unrestrained bees caged together and supplied a bulk source of sucrose solution containing a known quantity of *V. ceranae* spores [e.g., reference (34)]. Though both methods work well, each has potential downsides. Individual inoculation requires a much larger time investment to initiate the experiments, while group feeding studies may require greater numbers of replicates due to variance in volume and timing of consumption. Our data show that the novel soak method, based on the method used by Leonard et al. to inoculate bees with genetically engineered bacteria (14), is an efficient approach for achieving high-level infection in newly eclosed bees with minimal time input. When compared to the traditional individual feeding method, the soak method is not as precise in terms of dosing and likely delivers a higher spore dose. For the individual feeding method, each bee received ~4,000 spores essentially instantaneously. Neither bee weight changes nor solution volume depletion can be used to accurately determine the amount of spores consumed in the soak method as some solution remains on the exterior of the bee. However, for 65 bees, 1 mL of solution would allow for a maximum of 15.4 µL (containing 3 × 10^4^ spores) available per bee. Thus, the maximum number of spores consumed by bees in the soak method is 7.5× that consumed by bees using the individual feeding method. To determine the spores per bee for the group feeding method, we found an average consumption of 22.2 µL (±8.4 µL) of food consumed by newly emerged caged bees per 24 hours over the period from eclosion through 8 days post-emergence. Thus, these bees are consuming ~8 × 10^5^ spores over 2 days, demonstrating that a far greater number of spores are required in the group feeding method compared with either of the other two methods to achieve similar infection levels.

The efficacy of the soak method may be surprising based on the low propensity of bees of this age to respond to offered sucrose solution. The responsiveness of honey bees to sucrose solutions, as measured using the proboscis extension response [described in reference (35)], increases with age (36), and newly eclosed bees are considered to have very low sucrose responsiveness. In one study, it was found that only 22% of unconditioned newly emerged bees could reliably respond to a sucrose stimulus as compared to 74% of foragers (37). Additionally, newly eclosed bees are not known to engage readily in trophallaxis (38), although caged experiments have demonstrated that *V. ceranae* spores can be spread from older bees to newly eclosed bees via this route (39). The success of the soak method may instead be due to a different set of existing behaviors, namely nest cleaning or nestmate grooming, which are dominant activities of newly eclosed bees (40). *V. ceranae* is thought to be transmitted via a fecal-oral course in addition to oral-oral modes. Thus, the soak method may simulate a fecal-oral route of infection when young bees engaged in cleaning are exposed to spores that have been defecated in the colony. Current research suggests that *V. ceranae* infection does not induce hygienic behavior in bees (41, 42), so this activity is likely to be independent of the presence of spores in the solution although more research may be needed to confirm this assertion.

The original rationale for the study was based on the view that microsporidia have a dramatic reduction in the number of genes from the double-strand break repair pathways (8, 9). However, a recent study using genome-wide structural homology-based approaches found evidence that some of the previously unidentified DNA repair genes do, in fact, exist in microsporidia, although they encode proteins with highly divergent sequences (10). Despite the newly discovered presence of these genes, our evidence suggests a pronounced sensitivity of *V. ceranae* to host exposure to the DNA-damaging agent bleomycin. Another study found that the topoisomerase II inhibitor dexrazoxane reduced microsporidia proliferation in *Caenorhabditis elegans* (43). However, the mechanism of action through which this drug impacted microsporidia infection was unclear as several other topoisomerase II inhibitors acting through a different mechanism did not reduce microsporidia infection in this system. Assuming bleomycin is directly acting on *V. ceranae* cells (see below), our results may mean that these highly divergent DNA repair proteins are not as efficient as those from other eukaryotes. In addition, a recent study in yeast showed that the translesion DNA synthesis proteins, REV1 and REV3, are the most critical for recovery from DNA damage caused by bleomycin family compounds (44). To our knowledge, homologs of these proteins have not been found in microsporidia (8–10). Thus, *V. ceranae* and other microsporidia may still have a reduced ability to repair DNA damage, which fits with the original assumption that their reduced genomes confer reduced resilience at the cellular level.

Bleomycin might be expected to have negative impacts on host physiology and health [as recently described for the aminoglycoside paromomycin (13)] that could make this drug unsuitable for use in the field. We observed only moderate impacts on the survival of bees treated with bleomycin at the therapeutic doses of 1.25 and 0.613 µg/mL, with quite minimal effects found for 0.306 µg/mL for combined survival data. However, the finding of substantially reduced honey bee survival (>50% for 1.25 µg/mL and ~25% for 0.613 µg/mL) over the trial time period for one of the four trials (see Fig. S5 and Tables S1) suggests that a high degree of care is warranted regarding the use of this drug in the field. The significant induction for the previously characterized stress biomarker *l(2)efl* genes in the combined data also suggests that this drug may be unsafe for use in the field. Interestingly, we found that *UpdlC*, which encodes a honey bee UPD-like molecule (22), is upregulated by bleomycin treatment. Bleomycin-induced damage has been found to induce epithelial regeneration in other insects (12), driven in part by increased production and release of cytokines of the Upd family (27) [and reviewed in reference (28)]. These results suggest that in honey bees DNA damage promotes increased proliferation in part mediated by UPD production and activation of the JAK-STAT pathway. This pathway leads to midgut regeneration, which may provide tissue-level protection to the host in addition to the potentially increased resistance at the cellular level.

It is possible that bleomycin could reduce *V. ceranae* growth or survival via indirect effects by impacting DNA integrity in the honey bee host cells as opposed to direct effects on parasite cells. One mechanism could be through damaging host cells to an extent to which they are no longer able to fully support microsporidia growth. Sloughing of damaged cells has been observed in the fruit fly intestine after tissue damage (45), and sloughing of midgut epithelial cells has been observed in honey bees after exposure to other stressors, such as the thermal stress (22) previously shown to cause *UpdlC* induction. A second mechanism might involve a DNA damage-induced stress response that triggers immune pathways that may be detrimental to microsporidia growth (46), such as that found previously in the nematode (47). A third mechanism could be via effects on the microbiome. However, our results here indicate that bleomycin does not impact the honey bee microbiome at doses effective at reducing *V. cernaae* levels. These results stand in contrast to the recently described impact of aminoglycoside paromomycin treatment on the bacterial community of the microbiome (13). In the data reported here, we only examined the midgut (including the pylorus and half the ileum) and did not quantify bacteria microbiome levels in the hindgut potentially missing important effects. An additional caveat is that while the newly emerged bees used here did undergo natural eclosion with access to a frame for a few hours, we did not engage in other methods for bacteria inoculation. Thus, the bees used in this experiment did not have microbiome exposures that mimic natural colony inoculation, likely impacting their long-term microbiome (48). The authors of a previous study using a lepidopteran cell line model of *V. ceranae* infection to identify anti-*Vairimorpha* agents have also highlighted the critical point that any antibiotics would not be appropriate for use by beekeepers due to the restrictions on antibiotic use in honey bee colonies in many countries (49), again reducing the potential of bleomycin for use in the field.

Taken together, our data show that bleomycin can reduce *V. ceranae* levels at doses that do not present with high and consistent toxicity to bees. However, the data suggesting variable lethal and sublethal impacts on honey bees in these cage-based studies likely preclude pursuing bleomycin further for use in the field. Nevertheless, bleomycin may offer a useful tool in the research setting as a tool as a promising potential selection agent. Our cellular and molecular understanding of microsporidian infection has been limited by the complexity of their life cycle, the difficulties of culturing them, a paucity of model systems, and a lack of molecular tools [reviewed in reference (1)]. Novel molecular approaches for investigating microsporidia will be essential for revealing their complex biology. Methods to generate genetically modified microsporidia would represent an indispensable resource for making future progress (50). Elucidating appropriate selection agents is the first of several essential steps to reach this objective. Here, we show that bleomycin reduces *V. ceranae* infection levels and thus could be employed as a potential selection agent, although more work will be necessary to demonstrate the direct action of the agent on parasite cells. Future efforts to develop expression plasmids containing the relevant selectable markers will also be required. A gene conferring resistance to bleomycin and related compounds from *Streptoalloteichus hindustanus (ble)* (51) has been used as a selectable marker in a wide variety of cell types (52) and would be expected to confer resistance to bleomycin to microsporidia cells if suitable expression plasmids can be created. In addition, transformation techniques for delivering genetic material to these obligate intracellular eukaryotes must be established. Some critical progress has been made in culturing sporoplasm in *Nosema bombycis* for this purpose (53, 54), in delivering genetic material (55), and in generating stable transformants (56). Thus, the identification of bleomycin as having potent activity against this microsporidia species at doses that can be tolerated by host cells during selection would represent an important step in the efforts to develop tools for transforming microsporidia.

## Data Availability

All data supporting the findings reported here are contained within the article and supplemental material. Any further information is available from the corresponding author upon reasonable request.

## SUPPLEMENTAL MATERIAL

Supplemental material spectrum.03349-23-s0001.docx Figures S1 to S7 and Table S1.

ASM does not own the copyrights to Supplemental Material that may be linked to, or accessed through, an article. The authors have granted ASM a non-exclusive, world-wide license to publish the Supplemental Material files. Please contact the corresponding author directly for reuse.
