# Supplementary material for: Bleomycin reduces Vairimorpha (Nosema) ceranae infection in honey bees with some evident host toxicity
Source: Microbiol Spectr. 2024 Jan 5;12(2):e03349-23. doi: 10.1128/spectrum.03349-23 (PMC10846157; doi:10.1128/spectrum.03349-23)
Supplement: Supplemental material — Figures S1 to S7 and Table S1. [file spectrum.03349-23-s0001.docx]

**Supplemental Material**

**Supplemental Figures**

**Suppl Figure S1.** *V. ceranae* levels in midguts of newly eclosed bees inoculated via the soak method as determined by spore count using light microscopy on day 6, 14, and 18 dpi (A). *V. ceranae* levels in midguts of newly eclosed bees inoculated via the soak method in 30 separate trials as determined by spore count using light microscopy on day 10 dpi (B). *V. ceranae* levels in midguts of newly eclosed bees inoculated via the soak method and caged separately for the reminder of the experiment as determined by spore count using light microscopy on day 10 dpi (C). a ≠ b, p < 0.05

**Suppl Figure S2.** Representation of bleomycin structure (A). Survival of individual uninfected newly eclosed bees fed sucrose solution (n=102) or bleomycin at 100 (n=135), 25 (n=82) or 6.25 (n=144) µg / ml bleomycin starting on day 4 post-eclosion (B). Survival of individual uninfected newly eclosed bees fed sucrose solution (n=128) or bleomycin at 2.5 (n=114) µg / ml bleomycin starting on day 4 post-eclosion.

** Suppl Figure S3**. Western blot of midgut lysates from bees fed sucrose solution (C), or bleomycin at 2.5 or 25 µg / ml. Equal amounts of protein extracts were analyzed by western blotting with either pHistone H2AvD (Ser137) (top) or anti-GAPDH (bottom) antibodies.

**Suppl Figure S4**. Transcript levels of the *l(2)efl* genes *410087a* (B) and *724367* (C), as well as the cytokine *UpdlC* (C), relative to the β-actin in midgut tissue from landing board bees fed sucrose solution alone (n=4) or sucrose solution containing 2.5 (n=4) or 25 (n=4) µg / ml bleomycin for 2 day. Means ± SEM are shown and represent the expression values of the genes of interest, calculated using the 2(-ΔCT) method for individual bees. Statistical significance is noted as * for p < 0.05 and ** for p < 0.01.

**Suppl Figure S5**. Survival of individual uninfected newly eclosed bees fed sucrose solution (Trial 1 n=128, Trial 2 n=37, Trial 3 n=70, Trial 4 n=65, (n=142)) or bleomycin at 1.25 (Trial 1 n=144, Trial 2 n=430, Trial 3 n=69, Trial 4 n=72) (n=112), 0.613 (Trial 1 n=139, Trial 2 n=37, Trial 3 n=88, Trial 4 n=74) (n=112), or 0.306 (Trial 2 n=44, Trial 3 n=80, Trial 4 n=71) µg / ml bleomycin starting on day 4 post-eclosion for four independent trials.

**Suppl Figure S6**. Transcript levels of the l(2)efl genes *410087a* (A) and *724367* (B) and cytokine gene *UpdlC* (C) relative to the β-actin in midgut tissue from newly eclosed bees fed sucrose solution alone (Trial 1 n=10, Trial 2 n=8, Trial 3 n=8, Trial 4 n=8, Trial 5 n=8) or sucrose solution containing 0.613 µg / ml (Trial 1 n=10, Trial 2 n=8, Trial 3 n=8, Trial 4 n=8, Trial 5 n=8) for 4 day starting on day 6 post-eclosion for five independent trials. Means ± SEM are shown and represent the expression values of the genes of interest, calculated using the 2(-ΔCT) method for individual bees. Statistical significance is noted as * for p < 0.05 and ** for p < 0.01.

**Suppl Figure S7.** Levels of all bacteria (A) and the key microbiome community member *Gilliamella apicola* (B) as determined by qPCR in midguts of infected newly eclosed bees fed sucrose syrup containing 0.613 µg / ml bleomycin for 4 days. a ≠ b, p < 0.05. The results from 3 independent trials are shown.

**Supplemental Table S1**

**A. Survival Analysis for ‘Hi’ Lethal Doses**

| Log-rank (Mantel-Cox) test (recommended) |  |
| --- | --- |
| Chi square | 501.9 |
| df | 3 |
| P value | <0.0001 |
| P value summary | **** |
| Are the survival curves sig different? | Yes |

B**. Survival Analysis for ‘Hi’ Sublethal Doses**

| Log-rank (Mantel-Cox) test (recommended) |  |
| --- | --- |
| Chi square | 16.52 |
| df | 1 |
| P value | <0.0001 |
| P value summary | **** |
| Are the survival curves sig different? | Yes |

**C. Survival Analysis for all trials combined**

| Log-rank (Mantel-Cox) test (recommended) |  |
| --- | --- |
| Chi square | 16.52 |
| df | *1* |
| P value | <0.0001 |
| P value summary | ** |
| Are the survival curves sig different? | Yes |

**D. Survival Analysis for Trial 1**

| Log-rank (Mantel-Cox) test (recommended) |  |
| --- | --- |
| Chi square | 9.075 |
| df | 2 |
| P value | 0.0107 |
| P value summary | * |
| Are the survival curves sig different? | Yes |

**E. Survival Analysis for Trial 2**

| Log-rank (Mantel-Cox) test (recommended) |  |
| --- | --- |
| Chi square | 43.21 |
| df | 3 |
| P value | <0.0001 |
| P value summary | **** |
| Are the survival curves sig different? | Yes |

**F. Survival Analysis for Trial 3**

| Log-rank (Mantel-Cox) test (recommended) |  |
| --- | --- |
| Chi square | 0.6022 |
| df | 3 |
| P value | 0.8959 |
| P value summary | ns |
| Are the survival curves sig different? | No |

**G. Survival Analysis for Trial 4**

| Log-rank (Mantel-Cox) test (recommended) |  |
| --- | --- |
| Chi square | 6.171 |
| df | 3 |
| P value | 0.1036 |
| P value summary | ns |
| Are the survival curves sig different? | No |
|  |  |
|  |  |
